# Application of TOF-SIMS Method in the Study of Wetting the Iron (111) Surface with Promoter Oxides

**DOI:** 10.3390/molecules27030648

**Published:** 2022-01-19

**Authors:** Walerian Arabczyk, Jacek Rogowski, Rafał Pelka, Zofia Lendzion-Bieluń

**Affiliations:** 1 Department of Inorganic Chemical Technology and Environment Engineering, Faculty of Chemical Technology and Engineering, West Pomeranian University of Technology in Szczecin, 42 Piastów Ave., 71065 Szczecin, Poland; arab@zut.edu.pl (W.A.); Zofia.Lendzion-Bielun@zut.edu.pl (Z.L.-B.); 2 Institute of General and Ecological Chemistry, Lodz University of Technology, ul. Zeromskiego 116, 90924 Lodz, Poland; jacek.rogowski@p.lodz.pl

**Keywords:** TOF-SIMS, monocrystalline iron, surface (111), wetting, diffusion

## Abstract

In the present work, a simplified model of the Fe(111) surface’s promoter-oxide system was investigated in order to experimentally verify the previously proposed and known models concerning the structure and chemical composition of the surfaces of iron nanocrystallites in the ammonia-synthesis catalyst. It was shown that efficient oxygen diffusion from metal oxides to the clean Fe(111) iron surface took place even at temperatures lower than 100 °C. The effective wetting of the iron surface by potassium oxide is possible when the surface is covered with oxygen at temperatures above 250 °C. In the TOF-SIMS spectra of the surface of iron wetted with potassium, an emission of secondary FeOK^+^ ions was observed that implies that potassium atoms are bound to the iron surface atoms through oxygen. As a result of further wetting the iron surface with potassium ions, a heterogeneous surface structure was formed consisting of a thin K_2_O layer, next to which there was an iron-oxide phase covered with potassium ions. Only a limited increase in calcium concentration was observed on the Fe(111) iron surface upon sample annealing at up to 350 °C. As a result of wetting the iron surface with calcium ions, an oxide solution of CaO-Fe_x_O_y_ was formed. In the annealing process of the sample containing alumina, only traces of this promoter diffusing to the iron surface were observed. Alumina formed a solution with a passive layer on the iron surface and under the process conditions (350 °C) it did not wet the pure iron (111) surface. The decrease in Fe^+^-ion emission from the Fe-Ca and Fe-Al samples at 350 °C implies a reduction in the oxygen concentration on the sample surface at this temperature.

## 1. Introduction

The interactions of an iron surface with the hardly reducible oxides K_2_O, Al_2_O_3_, CaO have been the subject of many studies attempting to understand their influence on the elementary processes occurring on the surface of the iron catalyst used in the ammonia-synthesis reaction [1,2,3].

Based on research with AES (Auger Electron Spectroscopy) [4] and TPD (Temperature-programmed desorption) methods [5,6,7] that were carried out on models of monocrystalline iron samples under high vacuum conditions, it was found that potassium bonded directly to surface iron atoms that had completely desorbed from the iron surface at temperatures below 200 °C [8,9].

Further studies on the interaction of potassium with iron revealed that the FeOK structures on the surfaces of iron nanocrystallites were thermally stable due to the co-adsorption of potassium with oxygen [5,8,10,11,12], although the promoting effect of potassium on the nitrogen-adsorption rate was reduced [5]. It was shown that under the conditions of ammonia synthesis on the surfaces of single iron crystals, potassium could be stabilized in an amount corresponding to a maximum of 15% of the monolayer, which caused the reaction rate to double [12]. The highest increase in the rate of ammonia synthesis (approx. three times) on the polycrystalline iron foil was observed for the degree of surface coverage with potassium of θ_K_ ≈ 0.12 [13]. In addition, the effect of potassium oxide on the catalytic activity of iron increased in monocrystalline iron [12] and the iron catalyst [14] with the increasing ammonia pressure in the system.

Based on thermodynamic considerations, it was indicated [8] that the only stable form of potassium on the catalyst surface is KOH under the conditions of a hydrogen-ammonia atmosphere but decomposes under vacuum.

It was found [5] that when the iron surface is wetted with a two-dimensional K_x_O_y_ layer with a stoichiometry close to unity, the potassium and oxygen atoms can take adjacent positions on the iron [15] or potassium may be above the oxygen atom bound to the iron (“on the top”) [16].

A double-layer model was also shown [4,10], according to which promoters formed two monoatomic layers on the iron by wetting the entire surface of a catalyst. The first layer, in direct contact with the iron, was made of oxygen atoms. Promoter atoms were located above the oxygen. In the case of a doubly promoted catalyst, the surfaces of the nanocrystallites were wetted with a layer of the FeOK promoter (2D structure), and the nanocrystallites were connected to one another through the glassy phase of the promoters (3D structure). It was assumed that under the industrial conditions of ammonia synthesis and decomposition between two- and three-dimensional structures, there was a state of chemical equilibrium ensuring the stability of the catalyst structure. According to the model, the specific surface area of the catalyst was proportional to the number of oxygen atoms on the surface. Taking into account the size of the promoter ions, it was found that the surfaces of iron nanocrystallites can be covered with FeOK structures up to 50% [10,17]. Sites not occupied by promoters are capable of adsorbing nitrogen and ammonia molecules. Thus, in the double-layer model, promoters are not classified by their electronics or structure. It was shown that promoters that were considered to be electronic also affected the size of the specific surface area of the catalyst, which was only attributed to structural promoters. It was also shown that the geometric arrangement of the potassium atoms bound to iron via oxygen on the active surface is of greater importance than the electron-donating nature of this promoter.

The results consisted with the catalyst double-layer model were obtained by the TOF-SIMS (Time-of-Flight Secondary-Ion Mass Spectrometry) technique [18].

In this study, temperature-programmed TOF-SIMS (TP TOF-SIMS) was applied in order to study the diffusion of metal oxides on the iron (111) surface. Although this technique is not often used, it is possible to give examples of its application by other authors to the study of various processes such as the hydration of ammonia and hydrogen chloride on water-ice films at cryogenic temperatures [19], the properties and glass transition of amorphous acetic-acid films on Ni(111) [20], and the crystallization kinetics of thin water films on Pt(111) [21] and on bare or modified Si(100) and Si(100) surfaces [22]. Recently, TP TOF-SIMS has been used in studies on the desorption of self-assembled monolayers on gold substrates [23].

The aim of this work was to verify the double-layer model that was developed for the nanocrystalline-iron surface’s promoter-oxide system by testing the diffusion of promoter oxides using TOF-SIMS on the simple, well-defined system of an iron (111) surface covered with promoter oxides. A monocrystalline-iron sample with the (111) orientation was selected for the study. This was due to the fact that the highest reaction rate of ammonia synthesis was found for such an orientation of the iron surface [24,25,26,27].

In the present work, a simplified model system of Fe(111) was selected in order to experimentally verify the previously proposed models that were related to the structure and chemical composition of the surfaces of iron nanocrystallites in the ammonia-synthesis catalyst.

## 2. Result and Discussion

The idea for the experiment was based on the expected changes in the chemical composition of the analyzed area of the sample due to the diffusion of metal atoms from the metal-hydroxide-containing area of the sample surface, which was used as the source of a particular metal, to the selected area of the pristine Fe surface that was selected some distance away from metal hydroxide rich area.

In the first step of the experiments, the chemical composition of the surface of the samples was determined by TOF-SIMS. Table 1 shows the TOF-SIMS ion counts from the surfaces of the iron samples. It can be seen that rinsing in deionized water did not remove the residual amounts of Na, K and Ca from the surfaces of the iron samples.

In order to eliminate these contaminants, a TOF-SIMS depth profile was performed on the selected area of the sample surface. Application of the TOF-SIMS depth profiling allowed for the following of changes in the chemical composition of the surface during its sputtering, and this process was continued until the emission intensity of Na^+^, K^+^ and Ca^+^ ions decreased to constant values that did not change with prolonged sputtering time. After the depth profile was completed, the TOF-SIMS spectrum was collected from the sputtered area of the sample surface (crater). The number of counts of secondary ions emitted from the surface after Ar^+^ sputtering is shown in Table 1. After these preliminary cleaning procedures, the diffusion of potassium, calcium and aluminum on the sample surface was analyzed.

During the temperature programmed TOF-SIMS experiment individual ions diffuse over the passivated iron surface. The oxygen source was the passivated iron surface at the crater rim and the promoter-oxide source was remote from the analysis site. The concentration of promoters on the iron surface at the analyzed site changed depending on the diffusion rate, the indirect measure of which is the number of counts of secondary ions emitted from the analyzed surface initially cleaned by Ar^+^ sputtering. In the process, hydrogen is desorbed at temperatures below 100 °C. The relative arrangement of individual ions depends on the thermodynamic effects of their adsorption on a specific surface.

### 2.1. Wetting the Surface of Iron with Potassium Oxide

**Figure 1:**
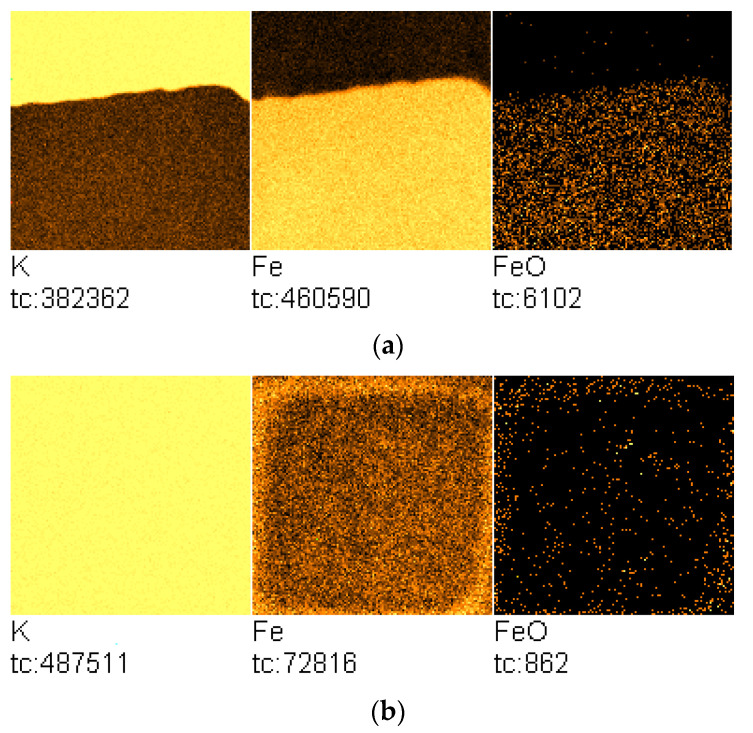
(**a**) TOF-SIMS image of the iron-sample surface with the visible region containing potassium oxide (field of view: 500 × 500 μm^2^; sample Fe/KOH); (**b**) TOF-SIMS image of the sample surface containing the area on which TOF-SIMS temperature-programmed profile was performed (field of view: 500 × 500 μm^2^; sample Fe/K-T). Total counts (tc) for particular ions are presented below each image.

Figure 1a presents the TOF-SIMS image of the sample surface with the visible boundary region between the area containing deposited potassium oxide and the pristine surface of the sample.

During the experiment, changes in the chemical composition of the initially clean iron surface were recorded using the TOF-SIMS technique. It was found that heating the sample induced an efficient potassium diffusion on the iron surface and resulted in a significant increase in potassium concentration at the initially clean Fe surface, obtained by argon sputtering, as shown in Figure 1b. The surface is heterogeneous and contains iron oxides in addition to the dominant potassium oxide.

The square region of the lower Fe^+^-ion emission corresponds to the sample surface area, which was cleaned by Ar^+^-ion sputtering prior to the TOF-SIMS temperature-programmed analysis.

Additional information on the changes in the structure and chemical composition of the sample surface during annealing was obtained using temperature-programmed TOF-SIMS analysis. The change in the intensity of iron ions is an indirect measure of the oxygen diffusion over the iron surface. Depending on the concentration of oxygen ions and promoters on the surface and the thermodynamic conditions, specific structures of iron, oxygen and potassium ions are formed on the iron surface.

During the TP TOF-SIMS experiment conducted on the Fe-K sample, the changes in the emission intensities of K^+^, K_2_O^+^ Fe^+^, Fe_2_O^+^, FeK^+^ and FeOK^+^ ions from the initially cleaned surface of the sample at different temperatures were studied.

Analysis of K^+^ and K_2_O^+^ ions provided direct information about the surface diffusion of potassium, while the signal of FeK^+^ ions was chosen as characteristic of the potassium-iron interaction on the sample surface. In addition, FeOK^+^ ions were selected to study the possible interaction between iron and potassium, including metal-oxygen bond formation.

**Figure 2:**
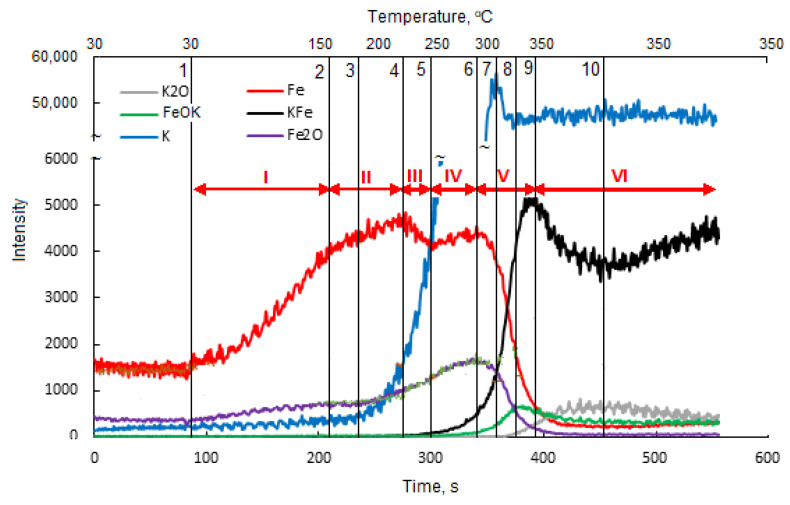
Temperature-programmed TOF-SIMS profile of the Fe-K sample.

Figure 2 presents TOF-SIMS profiles showing the emission of secondary ions from the area on the surface of the single iron crystal, which had been pre-cleaned by argon sputtering, depending on the annealing time and the temperature of the sample. The profiles can be divided into three areas corresponding to: 1. the iron surface covered with trace amounts of oxygen and potassium; 2. two-dimensional structures formed on the iron surface; and 3. the surface covered with a thin layer consisting of iron oxides.

The changes observed in the I scope of the profile (Figure 2) were attributed to the reconstruction of the amorphous iron surface in the area that had been pre-cleaned by ion sputtering, which took place as a result of annealing. The rate of the iron-surface reconstruction depends on the degree of surface deformation, temperature and time. According to the literature data, [28,29,30] bombardment of the surface with relatively low energy ions causes deformations to a depth thatoccurs in maximum of a few atomic layers. Therefore, even in the temperature range of 150–200 °C, we can expect a quick reconstruction of the surface deformed by ion bombardment, taking into account the small degree of the surface deformation.

It was assumed that in addition to the ordering of the structure of the analyzed surface area with the increase in temperature, an efficient diffusion of oxygen took place in this area, with the oxygen atoms diffused on the surface being located between the first and second layers of the iron atoms. [28]. As a result, despite the increases in the oxygen concentration and the emission of Fe_2_O^+^ ions (range II), a drop in the degree of iron oxidation determined by the ratio of Fe_2_O^+^ and Fe^+^ ion emission intensities (the Fe_2_O/Fe ratio) was observed. According to the literature data [28,29,30], two ordered structures with different oxygen contents are formed on the iron (111) surface: Fe(111) p(1 × 1)-O and Fe(111) (2√3 × 2√3)-30°-O, with the degree of surface coverage related to the number of layers of iron atoms on the surface (θ = 1 and θ = 2, respectively). The formation of two structures with an increasing Fe_2_O/Fe ratio is evidenced by inflections in the ion-emission profiles of Fe^+^, Fe_2_O^+^, FeO^+^ and Fe_2_^+^.

On the clean surface of iron, potassium atoms are only adsorbed at temperatures lower than 200 °C. A stable structure was formed with potassium on the Fe(111) p(1 × 1) and Fe(111) (2√3 × 2√3)-30° structures, with the maximum degree of oxygen coverage being 0.5 and 0.8, respectively. At higher concentrations of potassium, potassium oxide K_2_O is formed [30].

**Figure 3:**
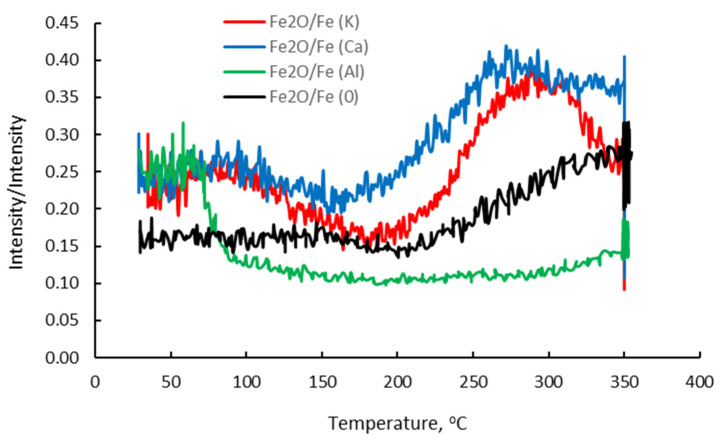
Changes in Fe_2_O/Fe ratio of the iron samples wetted with potassium, calcium and aluminum and the reference sample (without promoters).

Figure 3 shows the changes in the Fe_2_O/Fe ratio for the samples in which iron was wetted with potassium, calcium and aluminum and for the sample without promoters.

In area III, from point 4 to 5 (Figure 2), as the emission of K^+^ ions increased, the emission of Fe^+^ ions decreased. This proves the effect of suppressing Fe^+^ emissions by the surface structures of FeOK that begin to form in this temperature range. The effective formation of these structures with the temperature increase is clearly visible in area IV in Figure 2. The increase in the Fe_2_O/Fe ratio continues to point 8 (Figure 2 and Figure 3). For all the iron-containing ions in which potassium was absent, the characteristic maximum is visible in point 6. The potassium-containing ions have emission maxima that are shifted in time to each other in the following order: K^+^, FeOK^+^ and FeK^+^.

**Figure 4:**
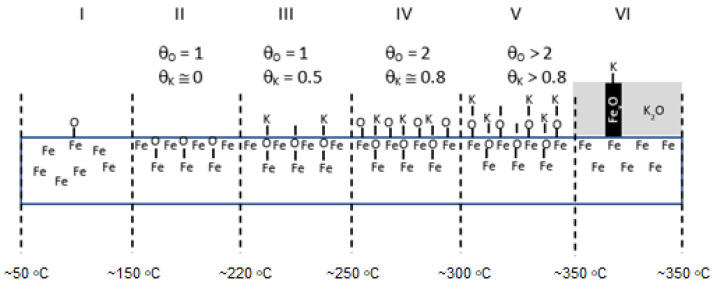
Schematic picture of surface structure of the Fe-K sample.

The sequence of shifts can be explained by the following processes. First, the phase nucleation of the three-dimensional structure of potassium oxide occurs as a result of combining oxygen from the iron surface with potassium. Stable structures of FeOK and FeK are formed as well. Finally, FeOK groups disappear from the surface. Above point 7, a K_2_O layer is formed on the iron surface with an increase in the Fe_2_O/Fe ratio and the potassium content (area VI in Figure 2, Figure 3 and Figure 4). Despite the formation of a potassium-oxide layer, the emissions of Fe^+^ and Fe_2_O^+^ ions are visible in the TOF-SIMS signal, similar to those observed at the beginning on the fresh, ordered iron surface covered with oxygen. This proves that a heterogeneous structure is formed, since next to the areas covered by potassium oxide there are areas of oxide iron Fe_x_O. The characteristic minimum emission of KFe^+^ ions occurring at point 10 with the simultaneous maximum K_2_O^+^ emission indicate the process of reducing the iron concentration on the two-dimensional iron structure in the initial stage of K_2_O-layer formation, and then increasing the amount in the iron-oxide phase. The resulting structures are shown schematically in Figure 4.

### 2.2. Wetting the Surface of Iron with Calcium Oxide

**Figure 5:**
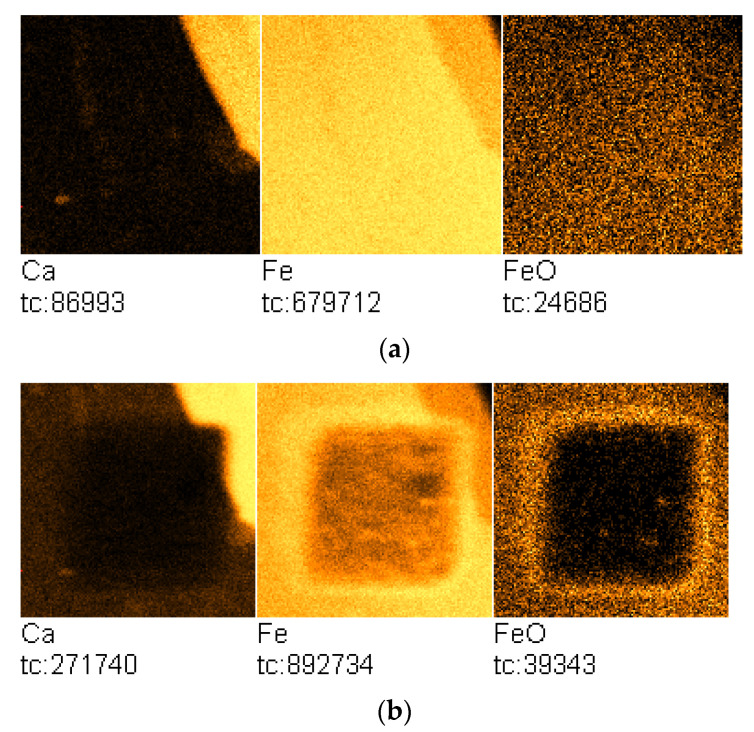
(**a**) TOF-SIMS image of the iron-sample surface with the visible region containing calcium oxide (field of view: 500 × 500 μm^2^; sample Fe/Ca(OH)_2_); (**b**) TOF-SIMS image of the sample surface area analyzed with temperature-programmed TOF-SIMS (field of view: 500 × 500 μm^2^; sample Fe/Ca-T). Total counts (tc) for particular ions are presented below each image.

Figure 5a presents the TOF-SIMS image of the sample surface with the visible region containing deposited calcium oxide.

The upper right parts of the Ca^+^ and Fe^+^ ion images in Figure 1a indicates the formation of a solution at the interface of two phases, since that area contains oxides of calcium and iron.

The TOF-SIMS image of the sample surface after annealing at 350 °C with a visible dark region corresponding to the analyzed region is presented in Figure 5b. In this case, the TOF-SIMS image does not show a discernible increase in Ca concentration in the analyzed area of the sample surface.

**Figure 6:**
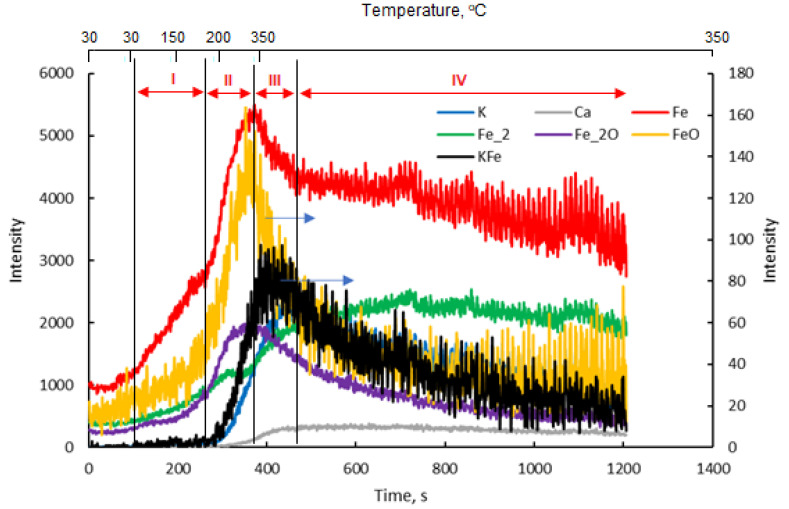
Temperature-programmed TOF-SIMS profile of the Fe-Ca sample.

After argon etching, the area of the transitional solution of calcium and iron oxides is also visible. The crater contains iron at varying degrees of oxidation. In order to obtain more detailed information concerning iron wetted with calcium, a temperature-programmed TOF-SIMS analysis was performed and the corresponding TOF-SIMS profiles are presented in Figure 6.

It can be noticed that during the initial part of profile corresponding to the constant temperature of the sample, the Fe^+^-emission intensity is constant. After that, when the programmed temperature begins to increase, the Fe^+^- and Fe_2_O^+^-ion-emission intensity raises. This increase in ion emissions was already observed after a small rise in temperature to around 50 °C. Similarly, the increase in Fe_x_^+^ and Fe_x_O^+^ emissions in the Fe-K sample at the initial step of the temperature increase can be attributed to the oxidation of iron by residual oxygen present on the sample surface.

The increase in the emission of Fe^+^ and Fe_2_O^+^ ions continued up to the sample temperature of about 300 °C, when a small increase in Ca^+^- and K^+^-ion emission took place. While calcium was intentionally introduced to the sample surface, potassium was a residual contaminant of the sample that could not be completely removed under the conditions of the current experiment. However, this result indicates that calcium and potassium can be simultaneously adsorbed onto the iron surface.

It is worth noting that the emission of Ca^+^ and K^+^ ions from the sample surface at temperatures above 300 °C was accompanied by the substantial signal from Fe^+^ and Fe_2_O^+^ ions, which are characteristic of the iron substrate. This is in contrast to the Fe-K sample, for which the surface layer formed by the diffusion of potassium caused the almost complete disappearance of the Fe^+^- and Fe_2_O^+^-ion emissions. This observation indicates that the thermally induced diffusion of potassium and calcium does not lead to the formation of a dense layer that inhibits Fe^+^- and Fe_2_O^+^-ion emission.

**Figure 7:**
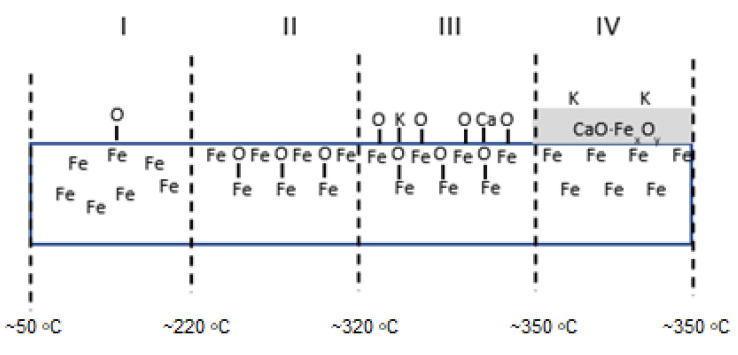
Schematic picture of surface structure of the Fe-Ca sample.

The TOF-SIMS profile of Fe-Ca and Fe-Al samples at the initial stages of annealing shows the efficient diffusion of oxygen on the sample surface, as evidenced by the increase in Fe^+^- and Fe_2_O^+^-ion emission (Figure 7 regions II and III). However, the shapes of the Fe^+^ and F_2_O^+^ profiles for the Fe-Ca and Fe-Al samples are different. The initial part of Fe^+^- and Fe_2_O^+^-emission profiles for the Fe-Ca sample is composed of two parts with different rates of Fe^+^- and Fe_2_O^+^-emission increases (II, III), with the first part (II) characterized by a slower increase in Fe^+^- and Fe_2_O^+^-ion emission than the latter (III). In contrast, the initial fast increase in Fe^+^ and Fe_2_O^+^ emission (II) from the Fe-K-sample surface is followed by the slower increase in the Fe^+^ and Fe_2_O^+^ signals (III).

In the Fe-Ca sample, similar to the Fe-K system, annealing at 350 °C resulted in the increase in the Ca^+^ and K^+^ emissions. Calcium oxide can dissolve in bulk iron; therefore, Ca^+^-and Fe^+^-ion emissions from the analyzed surface may be attributed to the formation of a surface layer containing mixed calcium and iron oxides of unknown stoichiometry. In contrast, potassium oxide does not form a solid solution in iron; hence, the observed K^+^-ion emission indicates presence of surface-bonded potassium. This assumption of potassium’s interaction with the iron surface is additionally confirmed by the observed emission of KFe^+^ ions at temperatures above 250 °C. In addition, the significant emission of Ca^+^ and Fe^+^ ions from the sample suggests that potassium does not form a continuous layer. A substantial K^+^ emission from the surface of the sample was detected despite the fact that potassium was present only in trace amounts as calcium hydroxide, which was used in this experiment as a calcium precursor.

### 2.3. Wetting the Surface of Iron with Aluminum Oxide

**Figure 8:**
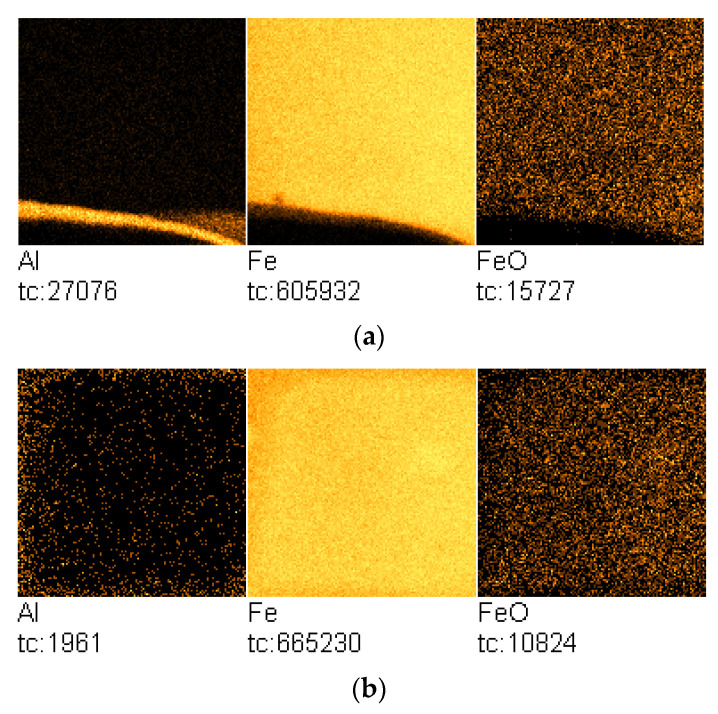
(**a**) TOF-SIMS image of the iron-sample surface with the visible region containing aluminum oxide (field of view: 500 × 500 μm^2^; sample Fe/Al(OH)_3_); (**b**) TOF-SIMS image of the sample surface area on which TOF-SIMS temperature-programmed analysis was performed (field of view: 500 × 500 μm^2^; sample Fe/Al-T). Total counts (tc) for particular ions are presented below each image.

TOF-SIMS images of the Al-Fe-sample surface area containing deposited aluminum oxide and the analyzed region after annealing at 350 °C are shown in Figure 8a,b, respectively.

The image in Figure 8b indicates that there was no wetting of the iron surface by the aluminum oxide, which is evidenced by negligible emission of Al^+^ ions from the analyzed surface.

The annealing process of the Al-Fe sample was further followed by time-resolved TOF-SIMS.

**Figure 9:**
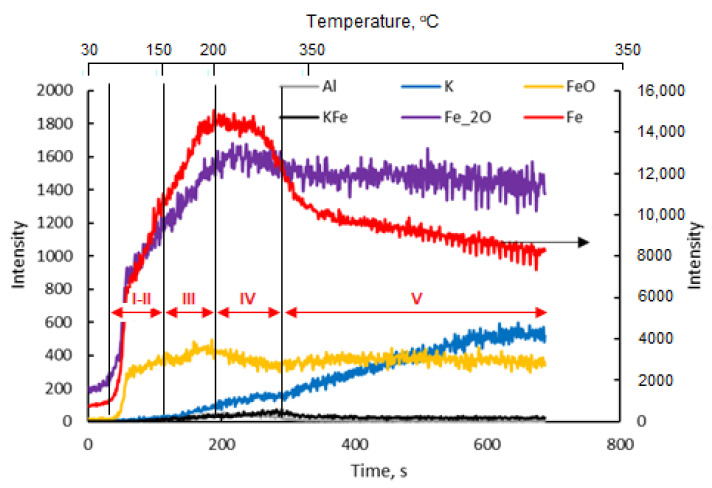
Temperature-programmed TOF-SIMS profile of the Fe-Al sample.

The temperature-programmed TOF-SIMS profiles for the Al-Fe sample in Figure 9 indicate that the increase in the sample temperature to about 50 °C resulted in a sharp increase in Fe^+^- and Fe_2_O^+^-ion emission, which can be attributed to the temperature-activated oxidation of iron.

**Figure 10:**
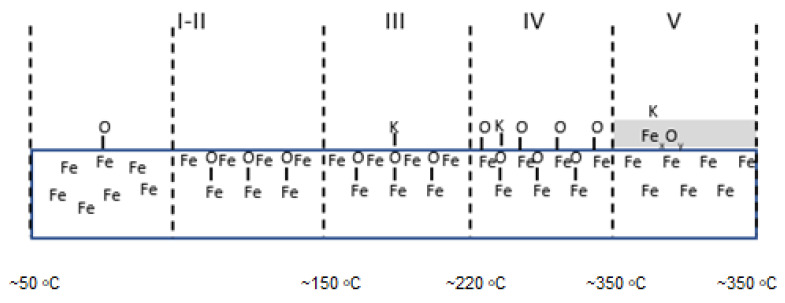
Schematic picture of surface structure of the Fe-Al sample.

In addition, the apparent change in the rate of the increase in Fe^+^-ion-emission intensity with increasing temperature suggests that there may be a change in the structure of the analyzed surface. The presumed change in surface structure is later depicted in Figure 10.

In contrast to potassium and calcium, no diffusion of aluminum on the iron surface was observed in the temperature range up to 350 °C.

TOF-SIMS profiles for the Fe-Al sample showed a similar sequence of processes to that observed for the Fe-Ca sample (Figure 10).

However, these processes took place at lower temperatures. The final structure of the sample surface after annealing at 350 °C was composed of potassium atoms bonded to iron in the subsurface layer of oxides Fe_x_O_y_.

The balance of the enthalpy of the iron-surface-wetting process with promoter oxides, ΔH_wet_, in relation to one surface bond can be expressed as follows:

ΔH_wet_ = −ΔH_form_ + ΔH_ads_ + (ΔH_Fe_^s^ − ΔH_Fe(MexO)_^s^) (1)

where ΔH_form_ is the sublimation effect from the bulk phase of promoter oxide, ΔH_ads_ is the promoter adsorption on the iron surface, and (ΔH_Fe_^s^ − ΔH_Fe(MexO)_^s^) is the change in the surface energy of the iron.

The literature provides divergent values of the surface energy of iron [31]. Based on data included in publication [32], the surface energy of the pure-iron surface was taken as ΔH_Fe_^s^ = −92 kJ/mol. It was also assumed that the enthalpy of the Fe^s^-O bond formation was the opposite of the enthalpy of breaking the bond in Fe_3_O_4_, i.e., ΔH_ads_ = −140 kJ/mol (in relation to a single bond).

The energetic effects of wetting the iron surface with promoters, (ΔH_wet_ + ΔH_Fe__(MexO)_^s^), were estimated based on Equation (1) and taking into account in the energy balance and the enthalpies of formation of the appropriate promoter oxide (in the case of potassium, ΔH_form_ = −182 kJ/mol in relation to a single bond). The estimated enthalpy of wetting the iron surface with the formation of FeOK groups is:

(ΔH_wet_ + ΔH_Fe__(K2O)_^s^) = 182 − 140 − 92 = −50 kJ/mol (2)

The values (ΔH_wet_ + ΔH_Fe__(MexO)_^s^) for calcium and aluminum are 232 and 269 kJ/mol, respectively.

According to the above values of the energetic effects of wetting the iron surface with promoter oxides, these oxides can be ranked in the following order when it comes to wetting ability:

Fe^s^-O-K > Fe^s^-O-H > Fe^s^-O-Al-(OH)_2_ > Fe^s^-O-Ca-OH

## 3. Materials and Methods

### 3.1. Sample Preparation

A single iron crystal with the (111) orientation and a purity of 99.98% was chosen for analysis. The sample was in the form of an elliptical cylinder with a height of 1.3 mm and the base axes were equal to 6 and 8 mm. The surface of the sample was polished with abrasive paper and then rinsed in deionized water. Aqueous solutions of KOH (pure for analysis, P.P.H. STANLAB Sp. J, Lublin, Poland.), Ca(OH)_2_ (guaranteed reagent for analysis, Merck, Warsaw, Poland) and Al(OH)_3_ (pure, POCH, Gliwice, Poland) were used as sources of K, Ca and Al on the Fe sample surface. In particular, to prepare individual samples containing potassium, calcium or aluminum, approximately 1μL of 1M aqueous solution of KOH, or the same amount of a saturated solution of Ca(OH)_2_ or Al(OH)_3_, was deposited onto the surface of the passivated samples. Such prepared samples were designated as Fe-K, Fe-Ca and Fe-Al, respectively. The area of the sample surface onto which the solutions of metal hydroxides were deposited had a diameter of approximately 2 mm. Under an ultra-high vacuum and elevated temperature conditions, the promoter hydroxides were dehydrated to the oxide form.

Directly before the experiment, a selected area of the sample surface chosen for metal-diffusion studies was cleaned by Ar^+^-ion sputtering. The area of the sputtered regions was 400 × 400 μm^2^ for the Fe-K and Fe-Al samples and 300 × 300 μm^2^ for the Fe-Ca sample. The centers of these sputtered regions were approximately 200 to 300 µm from the edge of the sample area where a drop of the metal-hydroxide solution was deposited.

### 3.2. TOF-SIMS Analysis

The secondary-ion mass spectra of the samples were recorded using a TOF-SIMS IV mass spectrometer manufactured by ION-TOF GmbH (Muenster, Germany). The instrument was equipped with a Bi liquid-metal-ion gun and a high mass-resolution time of flight-mass analyzer. Secondary-ion mass spectra were recorded from an approximately 50 × 50 μm^2^ area of the spot’s surface. During measurement, the analyzed area was irradiated with the pulses of 25 keV Bi_3_^+^ ions at a 10 kHz repetition rate and an average ion current of 0.2 pA. The primary-ion pulse duration was 1 ns. The analysis time was 30 s, which yielded an ion dose below the static limit of 1 × 10^13^ ions/cm^2^. The sputter gun was operated with Ar^+^ ions at a voltage of 3 keV. The current of the Ar^+^-ion beam used for sputtering was 20 nA. The temperature-programmed measurements were performer using the sample-stage heating and cooling systems of the TOF-SIMS IV apparatus. During temperature-programmed measurements the temperature was ramped at a rate of 1 K s^−1^.

## 4. Conclusions

The technologically important catalyst for the synthesis of ammonia has quite a complex system whose operation is influenced by many physical and chemical factors. This system has also been quite well researched so far (a double-layer model of the catalyst surface structure has been proposed). In the present work, a simplified model system of Fe(111) was selected in order to experimentally verify the previously proposed models related to the structure and chemical composition of the surfaces of iron nanocrystallites in the ammonia-synthesis catalyst.

It was shown that the efficient oxygen diffusion from metal oxides to the clean Fe(111) surface took place even at temperatures lower than 100 °C. The effective wetting of the iron surface by potassium oxide was possible at temperatures above 250 °C. The recorded emission of FeOK^+^ secondary ions implies that potassium atoms were bound to iron-surface atoms through oxygen. As a result of wetting the iron surface with potassium, a heterogeneous surface structure was formed, consisting of a thin K_2_O layer, next to which there was an iron-oxide phase covered with potassium ions.

Only limited increase in calcium concentration was observed on the Fe(111) iron surface upon sample annealing up to 350 °C. As a result of wetting the iron surface with calcium ions, a solution of CaO-Fe_x_O_y_ oxides was formed.

Aluminum formed a solution with a passive layer on the iron surface and under the process conditions (350 °C) it did not wet the pure-iron (111) surface. As a result of the annealing process, only traces of aluminum diffusing over the iron surface were observed.

The decrease in Fe^+^-ion emission from the Fe-Ca and Fe-Al samples at 350 °C implies the reduction of oxygen concentration on the sample surface at this temperature.

## Figures and Tables

**Table 1:** TOF-SIMS ion counts from the surfaces of the iron samples.

Sample	Analyzed Surface *	Number of Ion Counts				
		Al^+^	K^+^	Ca^+^	Fe^+^	Fe_2_O^+^
Fe-K	Fe	7732	6365	546	376,783	22,274
	Fe-Ar	102	3613	114	11,849	1059
	Fe/KOH	0	1,308,667	634	12,579	0
	Fe/K-T	134	1,312,932	1098	10,821	1628
Fe-Ca	Fe	11,759	3141	5397	431,777	26,706
	Fe-Ar	235	266	176	15,423	2844
	Fe/Ca(OH)_2_	0	25,785	41,468	4141	0
	Fe/Ca-T	579	14,597	3870	144,315	7022
Fe-Al	Fe	3053	5888	2827	234,881	17,048
	Fe-Ar	14	239	226	15,706	3674
	Fe/Al(OH)_3_	92,792	6526	0	100,752	9511
	Fe/Al-T	845	8993	1132	335,641	29,592

*: Fe—sample surface rinsed with demineralized water, Fe-Ar—surface sputtered with Ar ions, Fe/KOH, Fe/Ca(OH)_2_, Fe/Al(OH)_3_—sample surface containing deposited metal hydroxide, FeX-T—the primary Ar sputtered area of the sample surface after heating the sample to 350 °C.

## Data Availability

Not applicable.

## Sample Availability

The obtained samples are available from the authors.
